# Antioxidant properties, anti-nutritive and toxic factors of *Terminalia sericea* in Onderstepoort

**DOI:** 10.4102/ojvr.v91i1.2172

**Published:** 2024-10-28

**Authors:** Tirelo Matlala, Rejoice B. Maseko, Kedibone G. Kgosana

**Affiliations:** 1 Department of Chemistry and Chemical Technology, School of Science and Technology, Sefako Makgatho Health Sciences University, Pretoria, South Africa; 2 Chemistry Department, Faculty of Science and Engineering, University of Eswatini, Kwaluseni, Eswatini; 3 Department of Pharmacology and Therapeutics, School of Medicine, Sefako Makgatho Health Sciences University, Pretoria, South Africa

**Keywords:** anti-nutritive factors, toxic factors, feed, extracts, antioxidants

## Abstract

Anti-nutritive and toxic factors in plants adversely affect animal health and production. Hence, the aims of the study were to (1) carry out qualitative and quantitative analysis of these factors in *Terminalia sericea* that grows abundantly in Onderstepoort; (2) evaluate an affordable, efficient and reliable method that reduces these factors and (3) evaluate antioxidant properties of the phenolic compounds. Thus, sequential organic and aqueous extractions were carried out on *T. sericea* leaves and feed pellets. Extracts were subjected to qualitative analysis to confirm the presence of 11 phytochemicals. While quantitative analysis of nine phytochemicals was carried out on spectrophotometry, the phenolic extracts were further tested for antioxidant activity on 2,2-diphenyl-picrylhydrazyl (DPPH) assay where gallic acid (GA), quercetin (Q) and ascorbic acid (AA) were used as standards. The contents of GA, Q and AA in the phenolic extracts were determined on high-performance liquid chromatography (HPLC). Aqueous extraction was an effective method as most phytochemicals were extracted. Nine phytochemicals were qualitatively analysed in *T. sericea*. The highest contents of 1.97% and 2.37 ± 0.077 mg/g gallic acid equivalent with a significant difference (*p* < 0.05) were determined in oxalates and phenolics, respectively. Furthermore, 83.1% DPPH inhibition (IC_50_ = 0.0432 µg/mL) was determined relative to the standards (85.27% – 85.96%; IC_50_ = 0.000009 µg/mL – 0.00042 µg/mL). Subsequently, HPLC results showed that *T. sericea* contained high concentration of AA (62.5 ± 2.13 µg/mL) to confirm antioxidant properties of the leaves.

**Contribution:**

Though *T. sericea* leaves have antioxidant activities, the alkaloid contents may pose a threat to the livestock. Therefore, farmers around Onderstepoort should protect their livestock from feeding on these leaves.

## Introduction

Onderstepoort is a farming area that is dominated by many livestock farmers because of the attractiveness of the ecosystem and diversity of plant species that are often used as browse. Thus, livestock often obtain nutrition by feeding on tree leaves in the rangeland or browse, which has been cut by farmers as a feeding supplement because of its distinct nutritional values particularly during dry seasons. Because livestock rely on plants to maintain health and to enhance normal physiological processes, plants produce a wide range of phytochemicals such as secondary metabolites to protect themselves against herbivores, insects and pathogenic attacks and to survive abiotic stresses such as ultraviolet-B (UV-B) radiations and other adverse growing conditions (Vikram et al. 2020; Zaynab et al. 2018). Some of these metabolites include anti-nutritive factors (ANFs) and toxic factors or natural toxins that act as deterrents to their predators (Nte, Owen & Owuno 2023; World Health Organization [WHO] n.d.). Generally, ANFs are not highly lethal when ingested in small quantities; but in abundance, they may interfere with utilisation of nutrients from plants or plant products thereby depriving livestock of adequate nutrients. They include but are not limited to saponins, trypsin inhibitors, oxalates, phytates, lectins, cyanogenic glycosides, glucosinolates, S-methyl cysteine sulfoxide and gossypol (Kiranmayi 2014; Yankey et al. 2012). Conversely, toxic factors are compounds that are naturally produced by living organisms, which are not harmful to the organisms themselves but toxic to other organisms (WHO n.d.). Plant toxins are typical examples of toxic factors that fall under secondary metabolites including but not limited to cardiac glycosides, terpenoids and alkaloids.

Anti-nutritive factors of interest to this study such as saponins, tannins, oxalates and terpenoids are found in high concentrations in plant tissues and have a distinct way of exerting their anti-nutritive effects. For instance, saponins have detergent-like antimicrobial property that disrupts the membranes and inhibits the rumen microbial populations such as protozoa, methanogens, bacteria and fungi, which ferment feed by breaking down plant carbohydrates into volatile fatty acids and gases such as methane and carbon dioxide (Lallemand Animal Nutrition 2023; Patra & Saxena 2009). Several studies have demonstrated the detergent-like property of saponins that decreased protozoal numbers and fungi population in the rumen (Guo et al. 2008; Lu & Jorgensen 1987). Furthermore, Hess et al. (2004) reported that saponins increased the population of methanogens in sheep but decreased the methane production to indicate saponin interference with fermentation process.

Tannins are a diverse group of polyphenolic compounds found in the roots, wood, bark, leaves and fruits of most plant species that are often consumed as anti-nutritive agents in monogastric and poultry animals (Besharati 2022; Bocco 2017). They exert anti-nutritive effects by binding to and precipitating dietary proteins to form insoluble complexes that render proteins inaccessible for metabolism in the small intestines. Previous studies have showed tannins to have a strong affinity to proline-rich proteins in saliva of some animals where the resulting insoluble complex resisted both microbial or ruminant fermentation and enzymatic degradation (Hagerman & Robbins 1993).

Oxalic acids, on one hand, act as strong chelating agents to bind free cations such as sodium, potassium, magnesium and calcium to form soluble and insoluble salts where too much insoluble salts prevent mineral absorption (Adeniyi, Orjiekwe & Ehiagbonare 2009). Consequently, high oxalate contents in feed may lead to urolithiasis, a formation of calculi (stones) or concretions of mucus, protein and minerals in the urinary tract of ruminants (Mejia et al. 2022). On the other hand, terpenoids constitute a large proportion of the plant organic volatile compounds that are highly lipophilic and responsible for the odour in many plant species and fruits (Torres-Fajardo & Higuera-Piedrahita 2021). The odour intensity is dependent on the quantity of these molecules. Hence, their abundance and volatility play a significant role in deterring livestock feeding.

Otherwise, phytochemicals including alkaloids, quinones and cardiac glycosides have also been of interest to this study because they are likely to cause toxicity when consumed in abundance. Plant-produced alkaloids such as hepatotoxic pyrrolizidine, indolizidine, piperidine and tropane are of major importance in veterinary toxicology (Cortinovis & Caloni 2015). For instance, pyrrolizidine alkaloids are metabolised in the liver to the toxic pyrroles. As powerful alkylating agents, pyrroles react with cellular proteins and cross-link deoxyribonucleic acid (DNA) to cause cellular dysfunction, abnormal mitosis and tissue necrosis (Cortinovis & Caloni 2015). Additionally, quinones are highly reactive organic chemicals, which can act alone or generate reactive oxygen species (ROS), hydrogen peroxides and hydroxyl radicals in biological systems that are most likely to damage the DNA. Hence, their presence is often associated with immunotoxicity, cytotoxicity and carcinogenesis (Bolton et al. 2000). Furthermore, the presence of flavonoids may counteract the damaging effects of quinones as they are well known for their numerous pharmacological properties including antioxidant, antimicrobial, anti-inflammatory, antidiabetic, antimutagenic and hepatoprotective activities. While cardiac glycosides poisoning is a world-wide phenomenon, various plant species may be implicated particularly in the poisoning of cattle and sheep.

Nonetheless, many phytochemicals play a significant role in the treatment of various diseases and parasites because they have chemical structures that are unique from synthetic compounds. For instance, *Terminalia sericea* (hereinafter, *T. sericea*) Burch ex DC. is used in ethnoveterinary medicine for the treatment of various animal diseases because its phytochemicals have antimicrobial activities across various pathogens (Anokwuru et al. 2020).

Otherwise, *T. sericea* grows in Tanzania, Democratic Republic of the Congo (DRC), Angola, Namibia, Zimbabwe, Botswana and is naturally distributed in fewer provinces of South Africa such as Gauteng, Limpopo and North West (Anokwuru & Combrinck 2023; South African National Biodiversity Institute n.d.). The phytochemical contents of this plant species are, however, expected to vary enormously per geographical area as a result of factors such as environmental conditions and some plant-to-animal interaction relationships that occur in the ecosystem. These factors have a significant influence on plant health and growth. Hence, varying phytochemical contents of *T. sericea* were previously reported in Namibia and Limpopo province (Anokwuru et al. 2018; Shatri & Mumbengegwi 2020).

Based on previous studies, it is noteworthy that high contents of phytochemicals have potential to act as anti-nutritive or toxic factors. Hence, it is important to find relevant methods to reduce these components in browse or feed supplement. The current methods for reducing anti-nutritive and toxic factors include but are not limited to thermal processing, high energy electromagnetic radiations and other techniques (Kadam, Kumar & Kasara 2021). However, application of a single method has not been effective in reducing the anti-nutritive and toxic factors. Thus, a combination of several methods has been considered as the best strategy (Popova & Mihaylova 2019). However, because of lengthy multistep processes and high costs associated with application of several methods that often result in the quality of feed being compromised, many farmers in the sub-Saharan African countries are unable to keep apace as a result of poor infrastructures and limited resources.

Despite wide applications of *T. sericea* in ethnoveterinary medicine and the fact that the plant species bear fruits that attract livestock, particularly in areas such as Onderstepoort where it grows in abundance, there is no scientific information regarding its antioxidant property, contents of its phytochemicals with potential to act anti-nutritive and toxic factors when used as browse and a method that can be used to reduce the anti-nutritive and toxic factors from the browse. Hence, the aims of the study were to, (1) carry out qualitative and quantitative analysis of the selected anti-nutritive and toxic factors in *T. sericea* leaves found in Onderstepoort; (2) evaluate a simple, affordable, efficient and reliable method that reduces these factors and (3) evaluate antioxidant properties of the leaves.

## Research method and design

In order to achieve the aims of the study, the following methods and materials were considered.

### Plant collection

Permission to collect plant species around Gauteng province was obtained from the Department of Environmental Affairs. Thus, fresh leaves of *T. sericea* Burch ex DC. were collected in Onderstepoort (25° 38’ 52” South and 28° 10’ 54” East) communal area located in Gauteng province, South Africa between September 2020 and February 2021.The collected leaves were brought to Agricultural Research Council-Onderstepoort Veterinary Research (ARC-OVR) toxicology laboratory. The voucher specimen was prepared and deposited at the South African National Biodiversity Institute (SANBI) Herbarium for authentication.

### Materials

All analytical grade reagents and standards used in the study were purchased from Merck (Modderfontein, South Africa) and Inqaba Biotechnical Industries (Pty) Ltd. (Pretoria, South Africa). While Whatman No. 50 (24.0 cm) filter paper was purchased from Lasec (Midrand, South Africa). High-performance liquid chromatography (HPLC)-grade solvents were purchased from Monitoring & Control Laboratories (Johannesburg, South Africa). A bag of AFGRI Group Holding animal feed (bovine) (code R1153P) comprising crude protein, crude fibre, moisture, crude fat, calcium and phosphorus was obtained from the Agricultural Research Council stores and used as a negative control for analysis.

### Plant and animal feed processing

The collected leaves were washed with tap water, dried under air blowing fan at room temperature for 24 days and ground into powder using a rotor mill, ZM 200 (Retshch). Similarly, feed pellets were ground into powder using a rotor mill.

### Plant and feed extractions

#### Organic sequential extraction

The feed and plant leaf powders were weighed separately in triplicates in a ratio of 1:3 (weight/volume [w/v]) into organic solvents (hexane, acetone and methanol in this order) in sterile bottles. The mixtures were shaken vigorously at room temperature on a benchtop shaker (Labotec, model no. 202, Midrand, South Africa) for 16 h and filtered through Whatmann filter paper. The solid residues were dried and stored away. While the supernatants were collected and dried under reduced pressure before re-extraction with the next solvent, all dry extracts were used for analysis and then stored in a fridge at 4 °C.

### Aqueous extractions

#### Infusion

An inhouse laboratory procedure was followed. Twenty-five grams of feed and plant leaf powders were weighed separately in triplicates into a 500 mL beaker. An exact 10 g of baking soda (sodium hydrogen carbonate [NaHCO_3_]), 5 g of citric acid and 150 mL of warm water (55 °C – 65 °C) were added. Addition of sodium hydrogen carbonate and citric acid was to inactivate water hardening process because of excess divalent cations found in water. The mixtures were left on the bench for 30 min before being strained through a dry cheesecloth into a 250 mL conical flask. The solid residues were dried and stored away, while the supernatants were frozen in the –20 °C freezer. The frozen supernatants were lyophilised at 45 °C using VirTis benchtop SLC (SP Scientific) freeze dryer. These were used for analysis and then stored at room temperature (23 °C) afterwards.

#### Decoction

Twenty-five grams of feed and plant leaf powders were weighed separately in triplicates into a 500 mL beaker. Hundred and fifty millilitres of warm water (55 °C – 65 °C) were added into each beaker. The mixtures were boiled for 20 min at 80 °C – 100 °C, allowed to cool at room temperature, strained through a dry cheesecloth into a 250 mL conical flask. The solid residues were dried and stored away while the supernatants were frozen in the –20 °C freezer. The frozen supernatants were lyophilised at –45 °C using VirTis benchtop SLC (SP Scientific) freeze dryer. These were used for analysis and then stored at room temperature (23 °C) afterwards.

#### Extraction of phenolic compounds

The method was adapted from Proestos et al. (2005) with slight modification. A volume of 40 mL of 62.5% aqueous methanol and 10 mL of 6M (molarity) hydrochloric acid were added to 0.5 g dried sample. The mixture was stirred carefully, bubbled with nitrogen gas for 40 s – 60 s and refluxed in water bath at 90 °C for 2 h. This was cooled, filtered and the supernatant was collected, dried and used for analysis.

## Qualitative analysis of feed and plant leaf extracts

The phytochemical screening procedures were carried out as previously reported (Banu & Cathrine 2015; Babu et al. 2020; Kgosana 2019) with slight modifications. All tests were carried out in triplicate at ambient temperature unless otherwise stated.

### Sample preparation

One gram of hexane, acetone and methanolic crude plant leaf and feed extracts were completely dissolved in 14 mL of their extracting solvents. These were screened for the presence of several phytochemicals.

#### Test for terpenoids and sterols

*Salkowski test*: Five millilitres of each plant extract was mixed in 2 mL of chloroform followed by the careful addition of 1 mL concentrated sulphuric acid (H_2_SO_4_) and shaken. The red colour at the lower layer indicated the presence of sterols and the formation of yellow colour at the lower layer indicated the presence of terpenoids.

*Lieberman-Burchard test*: An amount of 0.05 g sample was dissolved in 2 mL of chloroform and a few drops of acetic anhydride and 2 mL concentrated H_2_SO_4_ were added. A purple colouration indicated the presence of triterpenes while bluish-green colouration indicated the presence of sterols.

#### Test for cardiac glycosides

*Liebermann-Burchard*: Five hundred milligrams of the dry extract was dissolved in 1 mL of acetic anhydride. The mixture was cooled on ice, and few drops of concentrated H_2_SO_4_ were added. A change from violet to a blue-green colour indicated the presence of steroidal nucleus (aglycone of the cardiac glycosides).

*Salkowski test:* Five hundred grams of dry extract was dissolved in 2 mL of chloroform. Then drops of concentrated H_2_SO_4_ were added to form a lower layer. A reddish-brown colour at the interface indicated cardiac glycosides.

*Keller-Kiliani test*: Two millilitres of plant extract was mixed with 750 µL of glacial acetic acid containing one drop of ferric chloride (FeCl_3_) solution, followed by the addition of 1 mL concentrated H_2_SO_4_. A brown ring forming at the interface indicated the presence of the deoxy sugar of cardenolides. A violet ring may appear beneath the brown ring; while in the acetic acid layer, a greenish ring may also form just gradually throughout the layer.

#### Test for saponins (foam test)

Six millilitres of water was added to 2 mL of extract in a test tube. The mixture was shaken vigorously and the formation of a persistent foam confirmed the presence of saponins.

#### Test for phenols (gelatin test)

Fifty grams of the extract were dissolved in 5 mL of distilled water and 2 mL of 1% solution of gelatin containing 10% sodium chloride (NaCI) was added to it. A white precipitate indicated the presence of phenolic compounds.

#### Test for flavonoids (alkaline test)

Two millilitres of solvent extract was treated with a few drops of 20% sodium hydroxide solution. The formation of an intense yellow colour indicated the presence of flavanols, flavones and chalcones.

#### Test for tannins

*Tannin*: One millilitre of an extract was added in a test tube. Exactly 3 mL of butanol-HCL reagent (95% of hydrochloric acid in n-butanol) was added. The test tube was plugged with cotton and placed on boiling water on a water bath for 60 min. A pink colour change indicated the presence of tannins.

*Vanillin-HCl test*: A few drops of vanillin-hydrochloric acid (1% vanillin/8% HCL in methanol [MeOH]) were added to 2 mL of the extract. Development of pink colour in the presence of tannins occurred because of conversion of phloroglucinol from catechin.

*Ferric chloride test*: Two drops of 5% ferric chloride were added to 1 mL of extract. The development of a dark bluish-black colour indicated the presence of tannins.

#### Test for alkaloids

The solvent extract was treated with 1 mL of 3N (normality) hydrochloric acid and filtered, and the supernatant was used to carry out the following tests.

*Mayer’s test*: A few drops of Mayer’s reagent were added to the filtrate and the formation of a reddish-brown precipitate indicated the presence of alkaloids.

*Wagner’s test*: A few drops of Wagner’s reagent were added to the filtrate and the formation of a pale yellow or cream white precipitate indicated the presence of alkaloids.

*Tannic acid test*: One to two drops of a 10% aqueous solution of tannic acid were added to 1 mL of the filtrate. An amorphous or crystalline precipitate confirmed the presence of the alkaloids.

#### Test for quinones

A small amount of extract was treated with concentrated 1 mL H_2_SO_4_ and observed for the formation of red colouration.

#### Test for protein

*Biuret test for proteins*: An equal volume of 40% NaOH solution and two drops of 1% copper sulphate solution was added to 0.5 mg of extract. The appearance of a violet colour indicated the presence of proteins.

*Xanthoproteic reaction test for proteins*: Two millilitres of an extract was warmed with nitric acid. Formation of yellow precipitates indicated nitration of an aromatic ring present in tyrosine, tryptophan and phenylalanine.

#### Amino acids (ninhydrin test)

About 2–5 drops of freshly prepared 1% ninhydrin reagent (in acetone) was added to 0.5 mg of extract and heated in boiling water for 1 min – 2 min. The formation of a purple colour indicated the presence of amino acids.

#### Oxalate test

Exactly 2 mL of the extract was treated with a few drops of glacial acetic. A dark green colour indicated the presence of oxalates.

## Quantitative analysis of feed and plant leaf extracts

The word ‘extracts’ in the procedures refers to the extracts of both plant leaf and feed. Apart from oxalate contents analysis, the extracts were analysed on a BioTek microplate reader, and the total contents were expressed in terms of ANF or toxin equivalent using the following formula:

$$C=\frac{cV}{M}$$ [Eqn 1]

Where *C* represents total content (mg/g) of test extract in ANF or toxin equivalent; *c* represents the concentration of ANF or toxin established from the calibration curve (mg/mL); *V* represents the volume of extract in mL; *M* represents the weight of extract in g.

### Oxalate contents

The method as described by McBride (1912) was used with slight modifications. Potassium permanganate (KMnO_4_) solution was prepared by dissolving 2 g – 4 g of KMnO_4_ powder in 400 mL of distilled water. The solution was transferred to a 100 mL clean burette. In order to standardise the KMnO_4_ solution, an exact sodium oxalate concentration of 0.15 molarity (M) was prepared. The solution was warmed to 70 °C – 90 °C and 20 mL was transferred into four clean 100 mL flasks. Titration was carried out with KMnO_4_ solution, and this was terminated immediately after a very faint pink colour that indicated an endpoint was reached. Then, molarity of KMnO_4_ was calculated based on the known concentration of sodium oxalate solution. Afterwards, all extracts were titrated with the standardised KMnO_4_ in order to determine the concentration of sodium oxalates.

### Total flavonoids

The method as described by Thangavel, Kasiramar and Sivakumar (2019) was used with slight modifications. Five millilitres of methanol was added into 25 mL centrifuge tube containing 0.5 g of each extract. The mixtures were shaken on a benchtop shaker (Labotec, model no. 202, Midrand, South Africa) at 100 revolutions per minute (rpm) for an hour, centrifuged at 3500 rpm for 10 min, and the supernatants were collected. Five hundred microlitres of each supernatant was transferred into a tube containing 1.5 mL of methanol. Then 100 µL of 10% aluminium chloride and 100 µL of 1 M potassium acetate were added. The mixtures were shaken with hand, and 2.8 mL of distilled water was added, left for 40 min at room temperature, and absorbance was read at 415 nm. The standard (quercetin) solutions (10 µg/mL – 100 µg/mL) were treated the same way as the extracts.

### Total phenolics

The method as described by Gopinathan, Rebido and Naveenraj (2014) was used with slight modifications. Five millilitres of methanol was added into 25 mL centrifuge tube containing 0.5 g of each extract. The mixtures were shaken on a benchtop shaker (Labotec, model no. 202, Midrand, South Africa) at 100 rpm, centrifuged at 3500 rpm for 10 min and the supernatants were collected. Five hundred microlitres of each supernatant was transferred into a tube containing 1.5 mL of methanol. Two and a half millilitres of a 10-fold diluted Folin-Ciocalteu reagent and 2 mL of 7.5% sodium carbonate were added to the mixture. The tubes were covered with parafilm and allowed to stand for 30 min at room temperature. Gallic acid (GA) solutions (30 µg/mL – 100 µg/mL) were prepared as standards and treated the same as the extracts. The absorbance was read at 715 nanometres (nm). The analysis was performed in three replicates.

### Saponins content

The method as described by Adusei et al. (2019) was used with slight modifications. Ten milligrams of each extract were dissolved in 5 mL of 50% aqueous methanol. Two hundred microlitres aliquots were transferred to test tubes together with 500 µL of 8% vanillin (in methanol) reagent and 500 µL of 72% sulphuric acid. The mixtures were placed on a water bath, which was set at 60 °C for 10 min and cooled on ice for 4 min. The absorbance was measured at 490 nm. Diosgenin solutions (10 µg/mL – 100 µg/mL) were prepared as standards and treated the same as the extracts. Total saponins contents were determined from the linear equation of a standard curve.

### Cardiac glycosides content

The method as described by Duffey and Scudder (1972) was used with slight modifications. The ouabain standard solutions was prepared in methanol (10 µg/mL – 100 µg/mL) while 100 mg/mL of each extract was prepared in their respective solvents. Three per cent solution of 3,5-dinitrobenzoic acid was mixed with 2 M NaOH in a ratio of 1:1. Hundred microlitres of the mixture was aliquoted into microwell plates and 10 µL of the standard solutions (10 µg/mL – 100 µg/mL), and the extracts were added. Absorbance was read at 450 nm.

### Tannins content

The total tannins from the extracts were determined by a slightly modified Folin and Ciocalteu method described by Tamilselvi et al. (2012). The sample was dissolved in methanol in a ratio of 1 mg/10 mL in a 15 mL centrifuge tube and made up to 20 mL. Two millilitres of 10-fold Folin-Ciocalteu was added together with 1 mL of 35% sodium carbonate. The solution was shaken and kept at room temperature for 30 min. Tannic acid solutions (0 µg/mL – 80 µg/mL) were prepared as standards and treated the same as the extracts. The absorbance was measured at 725 nm.

### Total alkaloids content

The method as described by Fazel et al. (2008) was used with slight modifications. One microgram of each extract was dissolved in 5 mL dimethyl sulfoxide (DMSO) and mixed with 1 mL of 2 N HCl. The mixtures were filtered and the supernatants transferred to separating funnels. Five millilitres of bromophenol blue, 5 mL of phosphate buffer (pH 4.7) and 5 mL chloroform were added and shaken vigorously. These were transferred into 25 mL volumetric flasks and diluted to the volume of volumetric flask with chloroform. The quinine standard solutions (10 µg/mL – 100 µg/mL) were prepared and treated the same as the extracts. The absorbance was measured at 470 nm.

### Terpenoid content

The method as described by Truong et al. (2021) was used with slight modifications. Two millilitres of each extract were added into a test tube and treated with 0.5 mL of acetic anhydride. The mixture was boiled for 2 min in a water bath then cooled sulphuric acid was added slowly before the absorbance was taken at 450 nm. Linalool standard solutions (20 µg/mL – 100 µg/mL) were prepared and also analysed the same as the extracts.

### Quinone contents

The method as described by Aja et al. (2015) was used with slight modifications. Fifty micrograms of each extract was soaked in 5 mL of 80% methanol and centrifuged for 5 min. One millilitre of the supernatant was transferred into three tubes containing 1 mL of 0.5% magnesium acetate and 1 mL of methanol. The mixtures were mixed very well, and the absorbance was taken at 515 nm. Alizarin standard solutions (10 µg/mL – 90 µg/mL) were prepared and analysed.

## Thin-layer chromatography and radical scavenging activity of the phenolic extracts

The free radical scavenging activity of the extracts was investigated according to a method described by Madaan et al. (2011). A stock solution of 2,2-diphenyl-picrylhydrazyl (DPPH) was prepared by dissolving 10 mg in 25 mL of methanol. Exact concentrations of 100 µg/mL of standard stock solutions (ascorbic acid [AA], GA and quercetin [Q]) were prepared by dissolving 10 mg in 100 mL of methanol. The solutions were used to make serial dilutions (2 µg/mL – 10 µg/mL) in methanol. Equal volume of different concentrations of standards was added to methanolic solution of DPPH, and the solutions were incubated for 30 min in the dark before the absorbance was measured at 517 nm.

A concentration of 100 mg/mL of each phenolic extract was serially diluted to lower concentrations (100 µg/mL – 1000 µg/mL) with methanol. Equal volume of different concentrations of phenolic extracts was added to methanolic solution of DPPH and incubated in the dark for 30 min at room temperature. After 30 min, the absorbance was recorded at 517 nm. Percentage radical scavenging activity was calculated using the following formula:

$$\%inhibition=\frac{\left[Ac-(As-Ao)\right]}{Ac\times 100}$$ [Eqn 2]

where *Ac* = absorbance of control (DPPH); *As* = absorbance of sample/ standard + DPPH; *Ao* = absorbance of sample/standard without DPPH interaction.

Chemical profiling of crude extracts was performed on thin-layer chromatography (TLC) using three different solvent systems, namely EMW (ethyl acetate : methanol : water) (40:5.4:5) (polar/neutral), CEF (chloroform : ethyl acetate : formic acid) (5:4:1) (intermediate polarity/acidic) and BEA (benzene : ethanol : ammonium hydroxide) (non-polar/basic) (90:10:1). Aluminium-backed TLC plates (Merck, 60 F254) were used. These were developed under saturated conditions with each of the three solvent systems namely. These were air dried in the fume cupboard and visualised under UV light (254 nm and 360 nm Camac Universal UV Lamp). Thereafter, 0.2% DPPH in methanol was sprayed on the dried plates to determine antioxidant activity. A colour change on chromatograms was observed over 30 min. 2,2-Diphenyl-picrylhydrazyl radical was reduced from a stable free radical (purple colour) to DPPH (yellow colour). The retention factor (Rf) for each active compound was calculated for each extract using the following formula:

$$Rf=\frac{\text{distance travelled by compound}}{\text{distance travelled by the solvent front}}$$ [Eqn 3]

## Analysis of phenolic extracts on high-performance liquid chromatography

High-performance liquid chromatography analysis was performed on an Agilent 1260 system equipped with a binary pump, high-performance degasser, high-performance autosampler, column thermostat and a variable wavelength detector (VWD) using a reversed-phase Eclipse XDB-C18 4.6 mm ID × 250 mm (5 µm) 80Å column at an ambient temperature. A flow rate of 1 mL/min was used at all instances. A concentration of 1 mg/mL stock solution of standards (AA, GA and Q) were prepared, and appropriate volumes from the stock solution were further diluted to prepare standards of varying concentrations. All the extracts were performed in quintuplicates. The extracts were also spiked with 50 µg/mL – 80 µg/mL of the standards to confirm the peak positions that are influenced by the matrix. Phenolic contents were determined using the standard curves and expressed in mg/mL unit.

### Statistical analysis

All measurements for quantitative analysis were carried out in triplicate, and the results were presented as mean ± standard deviation. An online Mann-Whitney U test was used to compare anti-nutritive and toxic factors in all the plant leaf and feed extracts and to prove the hypothesis that livestock feeding directly on the leaves of *T. sericea* acquire high anti-nutritive and toxic factors. While the null hypothesis was that livestock feeding directly on these leaves acquire the same or low anti-nutritive and toxic factors. The *p*-value was calculated at a significance level of 0.05. When the *p*-value was less than the significance level (*p* < 0.05), we rejected the null hypothesis and the result was considered significantly different.

### Ethical considerations

Ethical approval to conduct this study was obtained from the Sefako Makgatho Health Sciences Research Ethics Committee (SMUREC) (No. SMUREC/S/355/2022:PG).

## Results

### The effect of different extraction solvents on *Terminalia sericea* Burch ex DC

**FIGURE 1:**
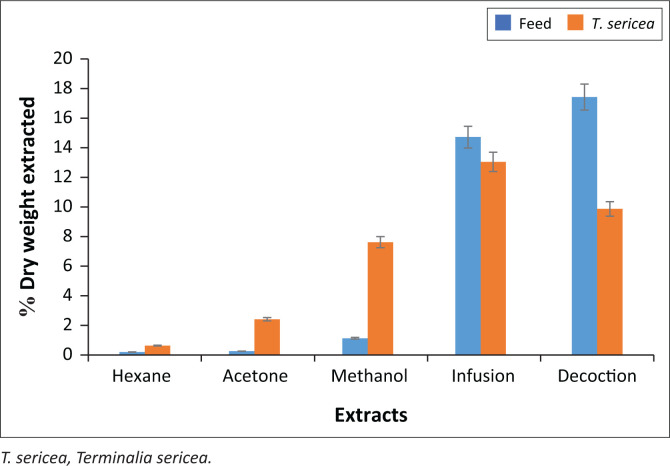
Extraction efficiencies of sequential organic and aqueous solvent systems in feed and leaves of *T. sericea*. The rectangular bars represent the mean values of the dry weight extracts in percentage while the standard deviations are represented as error bars.

The dry yields of various crude extracts explored in the study are illustrated in Figure 1. The aqueous solvents resulted in higher percentage yields of dry weight extracted as compared to the organic solvents. For organic solvents, the non-polar solvent (hexane) gave the lowest percentage yields.

### Qualitative analysis of the crude extracts

The presence of anti-nutritive and toxic factors was observed by colour changes or precipitation formation in different chemical reactions. Positive and negative test results were represented by (+) and (−) symbols, respectively. Nonetheless, the results revealed the presence of a wide range of phytochemicals. These include terpenoids, cardiac glycosides, saponin glycosides, phenols, flavonoids, tannins, quinones, proteins and amino acids in feed and *T. sericea* leaf extracts (Table 1).

**TABLE 1:** Qualitative test results of *T. sericea* leaf and feed extracts.

Phytochemicals	Test	Test results									
		H		A		M		I		D	
		Feed	*T. sericea*	Feed	*T. sericea*	Feed	*T. sericea*	Feed	*T. sericea*	Feed	*T. sericea*
**Terpenes**											
Terpenoids and sterols	Liebermann-Burchard	-	+	-	+	-	+	-	-	-	-
	Salkowski	-	-	-	-	-	+	-	-	-	-
Cardiac glycosides	Liebermann-Burchard	+	-	-	-	-	-	-	-	-	-
	Salkowski	+	-	-	-	-	-	-	-	-	-
	Keller-Kiliani	-	-	-	+	-	-	-	-	-	-
Saponin glycosides	Foam	-	-	+	-	+	-	-	-	+	-
**Phenolics**											
Phenols	Gelatin	-	-	-	-	-	-	-	+	-	+
Flavonoids	Alkaline	-	-	-	-	-	-	+	-	+	-
Tannins	Tannin	-	-	-	-	-	+	+	-	+	+
	Vanillin-HCl	-	-	-	-	-	+	-	-	-	-
	Ferric chloride	-	-	-	-	-	-	+	-	+	+
**Alkaloids**											
	Wagner	-	-	-	-	-	-	-	-	-	-
	Tannic acid	-	-	-	-	-	-	-	-	-	-
	Mayer	-	-	-	-	-	-	-	-	-	-
**Quinones**	Quinone	-	-	-	-	-	+	-	-	-	-
**Proteins**											
	Xanthoproteic	-	-	-	-	-	-	-	-	-	-
	Biuret	+	+	-	-	-	-	-	+	-	-
**Amino acids**	Ninhydrin	-	-	-	-	-	-	-	+	-	-
**Oxalates**	Oxalate	-	-	-	-	-	-	-	-	-	-

Note: +, dictated; -, not dictated.

H, hexane extract; A, acetone extract; M, methanolic extract; I, infusion; D, decoction; *T. sericea, Terminalia sericea*; HCI, hydrochloric acid.

Terpenoids and sterols, phenols, alkaloids, quinones, amino acids and oxalates gave (–) results in all feed extracts. Whereas *T. sericea* phytochemicals that were tested with more than one test procedure such as terpenoids and sterols, cardiac glycosides, tannins and proteins gave contradictory results as both (+) and (–) were recorded. Thus, contradictory results were recorded in; the organic extracts (hexane and acetone) of *T. sericea* when terpenoids and sterols were tested on two tests; in the hexane extract (feed) and acetone extract (*T. sericea*) when cardiac glycosides were tested on three tests; the methanolic extract (*T. sericea*), infusion (feed) and decoction (feed and *T. sericea*) when tannins were tested on three tests; the hexane extracts (feed and *T. sericea*) and the infusion (*T. sericea*) when proteins were tested on two tests.

In contrast, there was no (+) and (–) results when single test procedures were performed in triplicate on *T. sericea* leaves. Thus, the leaf extracts gave (–) results for saponin glycosides, flavonoids, alkaloids and oxalates tests. Aqueous leaf extracts gave (+) results for phenols while the decoction and an infusion gave (+) results for the tannins. Quinones gave (+) results in the methanolic extract. The amino acids were (+) in the infusion, whereas the proteins were found in the hexane and infusion.

### Quantitative determination of the anti-nutritive and toxic factors

All the calibration curves created were characterised by a high regression coefficient (0.9901–0.9986) indicating a good relationship between detector response (absorbance) and the tested concentration range (10 µg/mL – 100 µg/mL).

**FIGURE 2:**
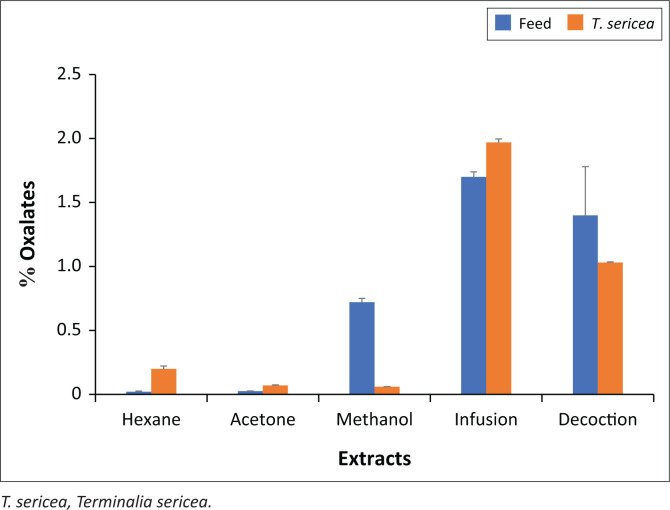
Oxalate contents in feed and *T. sericea* leaf extracts. The bars represent mean values of oxalate content in percentages while the standard deviations are represented as error bars. The null hypothesis was rejected, and the results were considered statistically significant (*p* < 0.05).

Oxalate contents were determined, and the results showed aqueous extracts to have high quantities (Figure 2). The highest oxalate contents that account to 1.97% and 1.7% were determined in the infusion of *T. sericea* and feed, respectively. This means that the infusion reduced most oxalates from the leaves. Therefore, the extracted leaves may not have anti-nutritive effects. Because of these high percentage yields, the infusion was considered as an effective and reliable method to reduce the oxalates in feed and *T. sericea*.

With respect to the organic extracts, *T. sericea* had relatively low oxalate contents that ranged between 0.2% and 0.06% to show that the respective methods were not effective and reliable in reducing the oxalates. The low contents obtained in these extracts simply show that higher oxalate contents were retained in the leaves.

**FIGURE 3:**
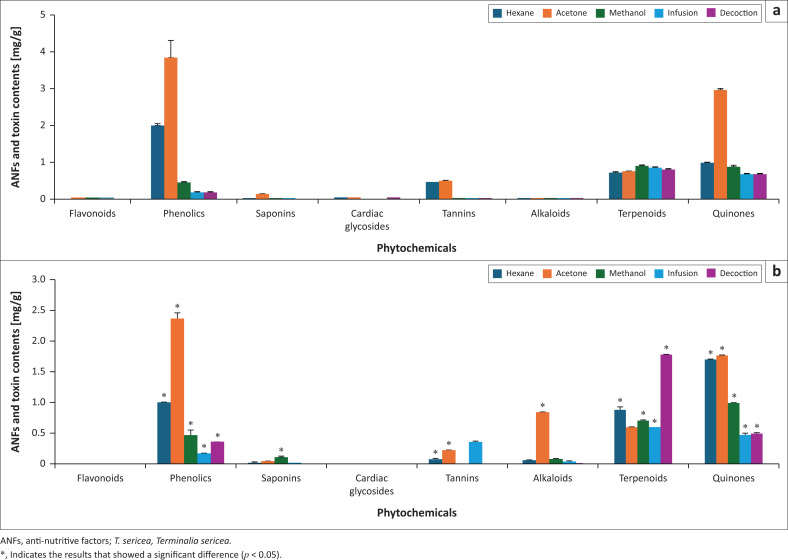
Anti-nutritive factors and inherent toxin contents of the extracts. (a) Feed extracts; (b) *T. sericea* extracts. The bars represent the mean values contents of the phytochemicals which may act as ANFs and inherent toxins while the standard deviations are represented as error bars.

Other phytochemical contents were determined in feed and *T. sericea* as illustrated in Figure 3a and b. With regard to an effective and reliable method to reduce anti-nutritive and toxic factors in the leaves of *T. sericea*, acetone extraction resulted in high phenolics (2.37 mg/g; *p* = 0.032), alkaloids (0.84 mg/g; *p* = 0.245) and quinone (1.76 mg/g; *p* = 0.007) contents to indicate extraction efficiency and reliability as compared to other extraction methods (Figure 3b). Apart from the alkaloids, these results had a significant difference (*p* < 0.05) to indicate that livestock feeding directly on these leaves acquire the same or low phenolics and quinones as putative toxic factors.

On the other hand, the decoction, infusion and methanol extractions showed high contents of the terpenoids (1.78 mg/g; *p* = 0.049), tannins (0.37 mg/g; *p* = 0.365) and saponins (0.116 mg/g; *p* = 0.095), respectively. Therefore, these extraction methods were found to be effective and reliable in reducing the respective anti-nutritive and toxic factors. Because the results for tannins and saponins had no significant difference, *T. sericea* leaves may have anti-nutritive effects to the livestock because of higher tannins and saponins contents. While the results for terpenoids had significant difference to show that their presence may not have anti-nutritive and/or toxic effects.

Furthermore, cardiac glycosides and flavonoids showed lowest content ranges of (0.0000017 mg/g – 0.0038 mg/g) and (0.00004 mg/g – 0.0078 mg/g), respectively. Even though the results of methanolic infusion and decoction extracts were not statistically significant, it is important to note that lower contents (< 0.1 mg/g) are often prone to errors. Thus, type I error was considered so as to reject the null hypothesis. This implies that livestock consumption on the leaves of *T. sericea*, regardless of being subjected to cardiac glycoside and flavonoid extractions, cannot have any toxic effects in the livestock.

With respect to the phytochemicals with high contents but showed no significant difference, type I error was considered where the contents were < 0.1 mg/g or when the feed content was high to reject the null hypothesis. For instance, the highest saponin content range in feed was 0.132 mg/g from the acetone extract. Therefore, the null hypothesis for the saponin content (0.116 mg/g; *p* = 0.095) in the methanol *T. sericea* leaf extract was rejected. Similarly, the acetone extract of feed had the tannins content of 0.49 mg/g; hence the null hypothesis of tannin content (0.37 mg/g; *p* = 0.365) in the infusion of *T. sericea* leaves was rejected. In contrast, neither type I error nor the high feed content (0.03 mg/g) could apply in rejecting the null hypothesis for the alkaloid content (0.84 mg/g; *p* = 0.245). Therefore, alkaloid contents in *T. sericea* may have toxic effects on the livestock.

### Thin-layer chromatography analysis, radical scavenging activities and high-performance liquid chromatography analysis of the phenolic extracts

**FIGURE 4:**
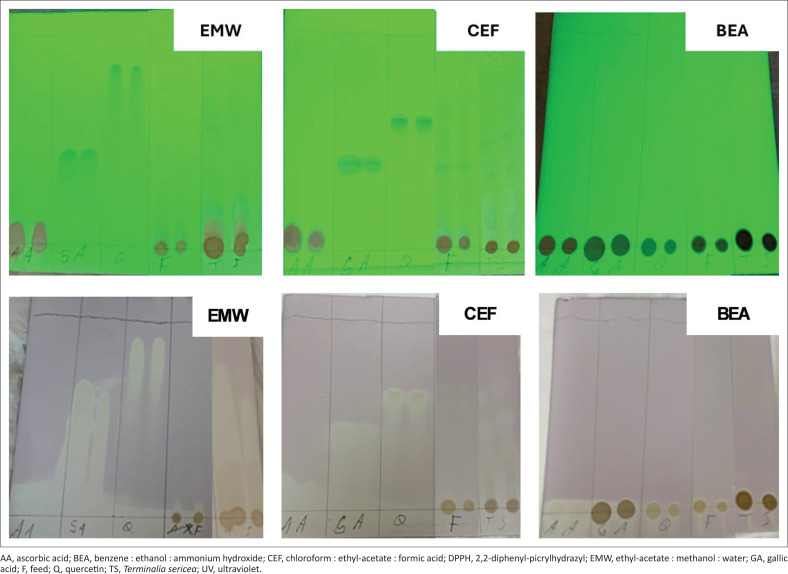
Chromatograms developed with EMW, CEF and BEA solvent systems. Green chromatograms show migration of compounds based on polarities taken under UV lamp. White chromatograms show plates sprayed with DPPH to determine antioxidant activities.

The green chromatograms (Figure 4) show the migration of compounds based on their polarities for the three positive control samples (AA, GA and Q) and the extracts (feed [F] and *T. sericea* [TS]). Although BEA chromatogram showed no migration of compounds shown as bands, EMW chromatogram showed migration of one compound for each spotted sample. In this chromatogram, Q bands migrated faster than all other bands. On the other hand, GA bands migrated faster than the bands of AA, F and TS. Whereas single compounds were only observed on positive control samples under CEF chromatogram. Because of stability, the two bands with Rf values of 0.63 and 0.7 in F were starting to fade away when the CEF photograph was taken. Nonetheless, the degree of polarity namely, *T. sericea* > feed > AA > GA > Q was noted as exhibited in the EMW chromatogram.

In terms of radical scavenging activities, all tested samples in BEA, CEF and EMW chromatogram showed the antioxidant activity at the spotted positions (Figure 4). The BEA chromatogram showed high antioxidant activities around the standards as observed from the intense whitish-yellow lining, which was less intense around feed and *T. sericea*. The intense lining indicated that the standards had high DPPH radical scavenging activities compared to the extracts. But then, the EMW and CEF chromatograms confirmed the presence of DPPH radical scavenging activities, which were also high in the standards and *T. sericea* extracts.

**FIGURE 5:**
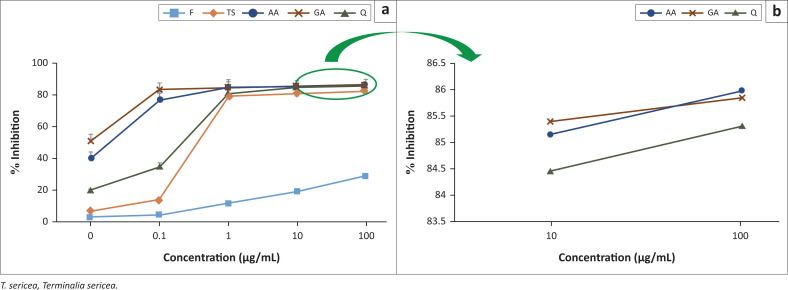
Percentage of 2,2-diphenyl-picrylhydrazyl (DPPH) inhibition versus concentrations of quercetin (Q), gallic acid (GA), ascorbic acid (AA), feed (F) and *T. sericea* (TS).

The antioxidant activities of all samples that were analysed spectrophotometrically and represented as percentage radical scavenging activity (% inhibition). The results indicated AA as the most efficient antioxidant with 85.96% inhibition at the concentration of 100 µg/mL (Figure 5). This was followed by GA (85.81%), Q (85.27%), T (83.1%) and F (29.45%).

Because all tested samples required a certain concentration to inhibit 50% of DPPH (IC_50_ values), the% inhibition data were used for that purpose. The results show that GA required the lowest concentration of 0.000009 µg/mL. While AA, Q, F and TS had IC_50_ values of 0.00034 µg/mL, 0.00042 µg/mL, 286 µg/mL and 0.0432 µg/mL, respectively.

**FIGURE 6:**
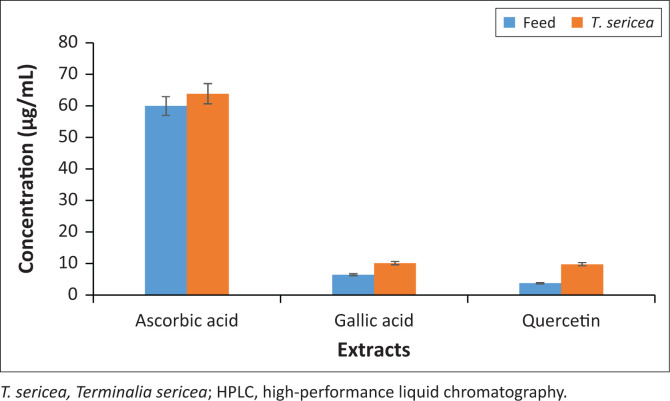
Concentrations of ascorbic acid, gallic acid and quercetin in feed and *T. sericea* phenolic extracts as determined on HPLC. The error bars represent the standard deviations.

Furthermore, the concentrations of GA, AA and Q from the phenolic extracts were analysed on HPLC. All calibration curves created were characterised by a high regression coefficient (0.9944–0.9961) to indicate a good relationship between detector response (peak areas) and the tested concentration range (10 µg/mL – 100 µg/mL). The concentrations corresponded with the result from the DPPH assay, which showed *T. sericea* with high radical scavenging activity (Figure 6). Thus, *T. sericea* contained high concentration of AA (62.5 ± 2.13 µg/mL) followed by GA (10.37 µg/mL ± 0.7 µg/mL) and Q (9.83 µg/mL ± 0.3 µg/mL).

## Discussion

### Extraction efficiency

The high percentage yields of dry weight extracted indicates that the aqueous methods were effective in reducing the anti-nutritive and toxic factors in *T. sericea* leaves. Interestingly, the infusion of *T. sericea* leaves gave higher yield than the decoction. This was expected as the decoction consists of abundant divalent cations such as calcium and magnesium that often form insoluble mineral deposits that complicate extraction efficiency of water (WHO 2011). Furthermore, the aqueous extraction methods do not have any implication on traditional healthcare practice because the practitioners often consider application of the cheap decoction but not the type of an infusion prepared from this study. The reason being that, traditional healthcare practice is dependent on the ancestor’s guidance, which frequently do not encourage adulteration of herbal medicines particularly with synthetic chemicals explored in this study. However, the infusion may have a positive impact on complementary medicine as it has been proven to yield concentrated secondary metabolites that may be useful for ethnoveterinary phytotherapy at lower costs.

### Qualitative analysis

Most phytochemical tests have various methods for a single ANF or toxin. However, selection of more than one method often gives rise to contradictory results where both (+) and (−) have been noted to imply that some tests are more sensitive than the others. Despite occurrence of contradictory results, all organic leaf extracts gave (+) results for terpenoids and sterols, while acetone extracts gave (+) result of cardiac glycosides on Keller-Kiliani test only. Additionally, the methanolic extract of *T. sericea* showed (+) result while all extracts showed (−) results as a confirmation to Shatri and Mumbengegwi (2020) previous report. It is worth noting that the (−) test results of oxalates, saponins and flavonoids could be attributed to sensitivity of the test method. Moreover, terpenoids and sterols, cardiac glycosides and tannins showed contradictory results that could neither be compared nor confirmed with the previous studies given that *T. sericea* had not been widely explored (Anokwuru & Combrinck 2023). For instance, this study explored Mayer, Tannic acid and Wagner tests for alkaloids that all showed the absence of alkaloids. However, Mayer, Dragendorff and Wagner were employed by Zhu, Shalom and Cock (2022) and gave the same results but resulted in contradictory results as reported by Babu et al. (2020). Otherwise, in order to rule out doubts on test results, more test procedures would be recommended.

### Quantitative analysis

The highest oxalate content of 1.97% was determined in the leaf infusion of *T. sericea*. This content cannot be regarded as lethal because it is around the range of oxalate content in feed that has gone through rigorous processing, monitoring, validations and optimisations for safe feeding. Although the oxalate content in *T. sericea* falls within the safe and acceptable level of less than 2% soluble oxalate of DM (dry matter) intake in ruminants; the leaves, however, may not be suitable for the non-ruminants because an acceptable soluble oxalate threshold level of < 0.5% has been recommended (Rahman, Abdullah & Wan Khadijah 2013).

The saponin contents were relatively low (0.0007 mg/g – 0.116 mg/g) in the leaf extracts and within the ranges of the feed (0.00032 mg/g – 0.132 mg/g). These contents are not expected to pose any form of threat to the livestock but expected to serve similar purpose as that of feed, which is to act as natural feed additives, anthelmintics and to manipulate ruminal fermentation and improve animal production (Gunun et al. 2019; Ramdani et al. 2023). Moreover, addition of saponins in feed as additives gained increasing interest because of the ban of most antibiotics from livestock feeding (Kholif 2023; Śliwiński et al. 2004). Generally, phytochemicals have been used to substitute chemical feed additives in fear of residues in animal products and the bacterial resistance to antibiotics (Patra & Saxena 2009). This purpose was shown in a previous study where feeding diets of lactating cows that were supplemented with 0.1 g/kg saponins from *Yucca schidigera* had no effect on performance (composition, milk yield and liveweight change) (Śliwiński et al. 2004). Because the supplemented content is almost equal to the highest content in *T. sericea,* they would be expected to have similar performance results on lactating cows.

Flavonoid contents of all the extracts were relatively low (< 0.1 mg/g). They were even > 24 times lower than the contents previously reported in 61 plant species with a range of 2.4 mg/g – 44.5 mg/g (Akhtar, Haq & Mirza 2015). In addition, Shatri and Mumbengegwi (2020) reported the highest total content of 207.5 mg/g in *T. sericea*. Perhaps the low contents in this study could be attributed to the plant subspecies, the geographical area of occurrence and the period of plant collection. In Shatri and Mumbengegwi (2020), the same plant species was collected in April 2016, in Namibia and 80% acetone/water were used as solvents for extraction. While in this study, plant collection was made between September 2020 and February 2021, at Onderstepoort where 100% acetone and the decoction (water) were used as solvents. Because the flavonoid contents in feed and *T. sericea* were almost within the same range, they are expected to serve the same purpose such as antioxidant, anti-inflammatory and anti-pathogenic activities.

Cardiac glycoside contents of all extracts were less than 0.1 mg/g. Hence, the leaves cannot be implicated in poisoning because only two cardiac glycosides were isolated from the genus *Terminalia* before 2001 (Zhang et al. 2019). Nevertheless, cardiac glycosides have both beneficial and toxic effects as they act as, (1) cardiotonic in low concentrations and (2) cardiotoxins in slightly higher concentrations (Nesy & Mathew 2014). Henceforth, the results from this study imply that the leaves possess cardiotonic effects.

By virtue of the leaf tannin contents (0.37 mg/g) being lower than the feed tannin content (0.49 mg/g), the null hypothesis was rejected to indicate that this content may not exert anti-nutritive effects to the livestock. Even though *T. sericea* had lower tannin contents than in feed, this was no surprise as small amount of tannic acids may be added in feed as an additive and dietary supplementation to reduce the oxidative damage of the membrane lipids, proteins and nucleic acid as a result of free radicals (Sultana et al. 2012). Otherwise, higher tannin content of 1.90 milligram quercetin equivalent per gram (mgQE/g) ± 0.03 mgQE/g was previously determined in the methanol/acetone extract of *T. sericea* leaves that were collected in May 2013 from Muyexe village (Giyani) in Limpopo province (Anokwuru, Ramaite & Bessong 2015). The solvents for extraction were 50% methanol in acetone and cold water. However, collection time, geographical area and solvents used for extraction were different in this study. Nevertheless, high tannin contents may be toxic and lead to death as they diminish nutrient utilisation.

Because the results showed that the alkaloid content (0.84 mg/g; *p* = 0.245) had no significant difference, this means that *T. sericea* leaves contain high alkaloids that may exert toxic effects. Although Shatri and Mumbengegwi (2020) reported a high content of 150 mg/g in these leaves, one study previously reported lower contents ranging between 0.176 mg/100 g and 0.3985 mg/100 g in some plant species such as *Ranunculus arvensis, Equisetum ravens, Carathamuslanatus* and *Fagonia critica* (Talema, Fedasa & Ketsela 2021). These contents were even way lower than this study’s alkaloid contents in feed that ranged between 0.01 mg/g and 0.03 mg/g. Otherwise, the phytochemical content variations could be attributed to biotic and abiotic factors during the period of collection. Or else, the low content in feed extracts could mean that the feed manufacturers selected the plant species with lower alkaloid contents for use in feed formulation.

The high terpenoid content (1.78 mg/g; *p* = 0.049) obtained in the leaf decoction, and the fact that the null hypothesis was rejected clearly indicates that *T. sericea* leaves are safe for feeding. Nevertheless, high terpenoid contents have potential to act as anti-nutritive and toxic factors as terpenoids interact with other organisms as antifeedants, repellents and toxins (Bohlmann & Keeling 2008). Nonetheless, the low contents in the organic extracts could be because of volatility property of volatile compounds, which are often determined with sensitive techniques such as gas chromatography–mass spectrometry (GC-MS) that give better extraction yields (Shang et al. 2002; Wang et al. 2008).

Though the phenolic content in feed extract had the highest content of 3.85 mg/g than the leaf extract (2.37 mg/g; *p* = 0.032), the results were significantly different; hence the null hypothesis was rejected to indicate that livestock feeding on these leaves may not acquire high phenolic contents. However, these values were low as compared to the previous study where the total phenolic content of 13.32 mg/g was reported in *T. sericea* leaves (Anokwuru et al. 2018). Conversely, Shatri and Mumbengegwi (2020) reported the highest total phenolic contents of 395.0 mg GA equivalent per gram (mgGAE/g) ± 0.08 mgGAE/g in this plant species. Whereas Anokwuru et al. (2015) reported the highest total phenolic content of 649.52 mgGAE/g in the hot water extract. These varying total phenolic contents could be because of the collection period, geographical area and both the environmental and laboratory conditions that differed enormously.

The highest quinone contents in feed and *T. sericea* were 2.98 mg/g and 1.76 mg/g, respectively. These results were shown to have a significant difference (*p* = 0.007) as an indication that *T. sericea* leaves are safe for livestock feeding. Moreover, the acetone extraction was considered as the most effective and reliable method of reducing the quinones in these leaves. Otherwise, quinones such as alizarin have been used for feed packaging because of their antioxidant, antimicrobial and antigenotoxic activities (Khan, Ezati & Rhim 2023). Even so, other studies showed that other quinones such as pyrroloquinoline quinone improve growth performance and provide safety to the livestock. For instance, Ming et al. (2021) reported that diets of weaned pig supplemented with 7.5 mg/kg of pyrroloquinoline quinone increased average daily gain and decreased feed conversion by 9.83% and diarrhoea incidences. The study also showed the increased activities of glutathione peroxidase, catalase and total antioxidant capacity in pigs. Therefore, based on the findings in this study, the low quinone contents in *T. sericea* can indisputably be regarded as safe to render some activities particularly those optimised for feed consumption.

### Thin-layer chromatography analysis, radical scavenging activities and high-performance liquid chromatography analysis

Thin-layer chromatography results indicated that the less polar compounds often move higher up the plate resulting in higher Rf values. For instance, the BEA chromatogram showed no migration of compounds to show the absence of non-polar compounds in all the tested samples and the fact that the inherent compounds were too polar that migration with this solvent system could be impossible. Nevertheless, EMW chromatogram showed that all extracts contained more polar compounds in relation to the standards and the trend TS > F > AA > GA > Q was observed. However, the trend could not be established on CEF chromatogram as the extracts showed migration of more than one compound.

Apart from quantitative and qualitative analysis, the study also determined antioxidant activities of *T. sericea* leaves. Antioxidant activities are primarily because of the presence of phenolic compounds including but not limited to the tannins and flavonoids. Therefore, it is important to note that the contents of phenolic compounds and free radicals must be kept in balance for the purpose of antioxidant activity. In an event where an imbalance occurs, these may lead to oxidative stress in the case of high free radicals (Sorelle, Ferdinard & Narcisse 2019) and anti-nutritive or toxic effect on the part of high phenolic compounds.

Nonetheless, the results of the TLC plates subjected to radical scavenging activity showed activities to indicate the health beneficial effects of these leaves. Interestingly, the determined percentage inhibition corresponded with the intensity of white linings observed on the chromatogram. As compared with the chromatogram, low percentage inhibition (29.45%) was expected in feed as opposed to *T. sericea* (83.1%) and the standards (85.27% – 85.96%). Moreover, *T. sericea* had the IC_50_ value of 0.0432 µg/mL to show its efficiency as a radical scavenger. This result does not differ much from the previous study where radical scavenging activity with IC_50_ value of 57.68 µg/mL was reported from the methanol/acetone extract of *T. sericea* leaves (Anokwuru et al. 2015). In addition, Anokwuru et al. (2018) reported a lower IC_50_ value of 6.44 µg/mL to show variation of quantities of the phenolic extracts.

Furthermore, the study reported on the concentrations of AA, GA and Q to assess the free radical active components of the phenolic extracts. Ascorbic acid gave the highest concentrations in both feed (60 µg/mL) and *T. sericea* (62.5 µg/mL) extracts. This was not surprising because AA is often supplemented in feed to reduce oxidative stress, improve animal health and increase growth performance because of its anti-inflammatory and antioxidant effects (Gan et al. 2018; Hieu et al. 2022). Furthermore, despite other antioxidants that may be found in *T. sericea*, AA may be considered dominant in neutralising the DPPH free radicals so as to become a less reactive free radical itself. Because termination of a free radical may be done through neutralisation by another antioxidant or with other mechanisms (Best 2024), the presence of GA, Q and other antioxidants in *T. sericea* may serve the purpose.

Conversely, the low contents of GA were expected in feed because it has gone through rigorous monitoring during the manufacturing processes before it was confirmed and considered acceptable for use as an additive and nutritional supplement. This was done on the basis that a substantial amount of GA can potentially contribute to significant toxicity (Read et al. 2019).

### Strengths and limitations

The selected time period and the geographical area for collection of plant species were the limitations to this study. In the terms of the strengths, the study used HPLC analytical instrument as an ideal technique known to analyse data with high accuracy and precision.

### Implications or recommendations

Despite the antioxidant properties, livestock feeding on the leaves of *T. sericea* are deemed to be at risk of poisoning because of high contents of the terpenoids and alkaloids. Again, high contents of tannins and oxalates have detrimental anti-nutritive effects, which may lead to death in worse-case scenarios. With respect to the choice of an effective, safe, affordable and reliable method to reduce the anti-nutritive and inherent toxic factors in *T. sericea* plant leaves, the infusion proved to be the best option as it satisfied all these requirements. Moreover, this method may also preserve some nutrients that are sensitive to high temperatures.

## Conclusion

The methods used in the study were selective in reducing the phytochemicals in *T. sericea* leaves. Because of safety associated with organic extraction (hexane, acetone and methanol), the aqueous extraction (infusion and decoction) was considered as a simple, affordable, effective and reliable method to reduce the oxalates, tannins and terpenoids. Apart from the alkaloids, the results were significantly different to show that livestock feeding directly on these leaves acquire the same or low anti-nutritive and toxic factors. Moreover, the leaves had antioxidant properties.
